# Papal and Talmudical Opinions on Craniotomy

**Published:** 1886-01

**Authors:** 


### Editorial.

### PAPAL AND TALMUDICAL OPINIONS ON CRANIOTOMY.

A few days ago the daily papers reported from New York that every Roman Catholic physician in that city was in receipt of a circular containing a declaration by the Pope that the killing of an unborn infant is never right, even when the life of a mother can be saved in no other way. This bears upon the surgical operation known as *Craniotomy*, which is generally practiced by the profession in cases where a choice is forced between losing two lives and saving one by hastening the death of the other. Inquiry of Bishop Corrigan, in New York, elicited the fact that the letter to the physicians was not official, but that the statement of the Pope's ruling was in itself correct. The question had been discussed carefully by the Inquisitors-General at Rome, who voted unanimously against the practice, on the ground that the taking of a human life could not possibly be justified, except as a legalized punishment for crime. This finding was formally referred to the Pope, who ratified and promulgated it a few weeks ago.

It may be of interest to learn the Rabbinical view on this question. In Mishna *Oholoth*, vii. 6, the undisputed decision is laid down that "in case of a dangerous parturition, it is justifiable to kill the unborn infant in order to save the mother, as her life precedes its life."

The above paragraphs, taken from the *American Israelite* of recent date, give, it is presumed, the opinion of the large classes of our population—the Roman Catholics and the Jews—upon the subject indicated.

With medical men, this question is no longer in dispute. Craniotomy is one of the recognized resources of art; its propriety and duty are admitted. The conditions which demand it, and the manner of performing it, are clearly defined. Unconscious that it was ever the subject of religious disputation, the profession has universally adopted the Rabbinical view of it as being most in accordance with common sense and with the highest regard for the safety and sanctity of human life.

Napoleon solved this doubt for the persons in attendance on the difficult accouchment of the Empress. It seemed as if the mother or the child must be sacrificed. The alternative was submitted to the Emperor. "Save the mother, though the child perish," was his prompt reply. "Treat the Empress as you would the wife of a peasant."

The opinion of physicians of the propriety and necessity of this operation in the cases in which it is applicable is universally recognized, but it is questionable whether the common practice of it has not tended to demoralize public thought in tolerating the general crime of destroying the life of the unborn.

It is easy to argue that if it is justifiable, at the full term of pregnancy, to kill the child to save the mother, it is also pardonable, at any earlier period of gestation, to destroy the fetus to shield the mother from shame, disgrace or suffering. Such reasoning tends to familiarize the mind with crime, to obliterate the instinctive horror of child murder, and to destroy all the restraints which nature, humanity, religion and law have erected for the protection of helpless infancy.

Whatever the cause, the fact is admitted that infanticide is in our country a growing evil. It has become a common topic of conversation amongst all classes of people, and is regarded as a possible, though illegal, expedient within the reach of those who seek it.

The rigid rule of the Catholic Church, that the taking of human life cannot possibly be justifiable, except as a punishment for crime, enforced by the influence of religion and conscience, would greatly tend to the lessening of this enormous evil. The census tables of this and other countries abundantly establish this position. But without going to this extreme, the medical profession can do much to stay the current of child-murder, which gathers strength annually as it runs over the land. They can confine the operation of craniotomy to the rare cases to which common sense and professional experience restrict it, and by denouncing in unmeasured terms the horrible crimes of abortion and infanticide, which are thought by some to be excused by it.

